# Holo-omics analysis reveals the influence of gut microbiota on obesity indicators in Jinhua pigs

**DOI:** 10.1186/s12866-023-03011-8

**Published:** 2023-11-03

**Authors:** Shuang Liu, Xueshuang Lai, Qinqin Xie, Zhen Wang, Yuchun Pan, Qishan Wang, Zhe Zhang

**Affiliations:** 1 College of Animal Sciences, Zhejiang University, Hangzhou, 310030 China; 2 School of Agriculture and Biology, Department of Animal Sciences, Shanghai Jiao Tong University, Shanghai, 200240 China

**Keywords:** Jinhua pigs, Multi-omics associations, Obesity, Lnc RNA, 16 s rRNA

## Abstract

**Background:**

The mechanisms behind obesity are complex and multi-faceted, involving the interplay of both host genomics and gut microbiome. In recent years, research has largely focused on these factors separately, but rarely from the viewpoint of holo-omics, which considers the host and microbiome as an integrated entity. To address this gap in knowledge, the present study aimed to investigate the holo-omics basis of obesity in Jinhua pigs, a Chinese indigenous breed known for its high degree of fat deposition and superior meat quality.

**Methods:**

Six pigs with extreme obesity phenotype were selected from a larger cohort of eighteen Jinhua pigs, and the contents of the jejunum, cecum, and colon regions were collected after slaughter at 240 days of age. The data obtained was processed, denoised, and annotated using QIIME2, with expression differences being analyzed using edgeR software.

**Results:**

The results showed significant differences in jejunal microbial diversity and composition between the two groups, with gut transcriptomics also indicating that differentially expressed genes in the jejunum were enriched in lipid metabolism pathways. These findings provide further evidence of the influence of the gut microbiome and host gene expression on fat deposition in Jinhua pigs.

**Conclusions:**

This study provides valuable insights into the mechanisms of fat deposition in Jinhua pigs from the viewpoint of holo-omics. The integration of host transcriptomics and microbiome data helps shed light on the complex interactions between the host and gut microbiome, and highlights the importance of considering both factors in our understanding of obesity.

**Supplementary Information:**

The online version contains supplementary material available at 10.1186/s12866-023-03011-8.

## Background

The gut microbiome has been shown to play a significant role in host energy absorption and storage [1], and the composition and functionality of the microbiome have been implicated in various host traits or diseases, including obesity [2–4]. Hosts and microbes exhibit a symbiotic relationship, with many microbial genes being shared among individuals forming a "core microbiome." This suggests that the interplay between host and microbiome contributes to host phenotypic indicators [5]. For example, previous studies have revealed similar microbial community structures in hereditary obesity populations, with changes in bacterial diversity and metabolic pathways associated with obesity phenotypes [6].

While mice are frequently used as animal models in obesity research, the results obtained are not easily translated to humans due to physiological differences [7]. Pigs, on the other hand, are considered a more appropriate biomedical model for human metabolism and obesity research due to their similar metabolic characteristics and organ size. In addition, the structure of the pig gut microbiome is more similar to that of humans [8–10].

After being domesticated by humans since ~ 7000 years ago, many pig breeds with different features has been formed around the world. For instance, commercial western pig breeds have been selected to grow faster and provide more lean meat, while Chinese pig breeds are known for their high fat content and strong stress resistance. A previous study performed a comparison of microbiota in different intestinal segments between Jinhua pigs (a Chinese indigenous pig breed with high propensity for adipogenesis) and a European commercial pig breed, Landrace, using 16S rRNA gene sequencing, and shown that the bacterial communities in duodenum, jejunum and cecum are different between the two pig populations [11]. Therefore, it is reasonable to hypothesize that gut microorganisms can explain the phenotypic differences between Chinese and western pig breeds. In addition, the microbial contribution to the obesity phenotype can differ between gut segments. A previous study investigating the relationship between intestinal microbes and obesity phenotypes in pigs showed that cecum and colon microbes contributed more to body weight and average daily weight gain, ranging from 22 to 37%. In contrast, the contribution of jejunum and cecum microbes to backfat thickness and intralipid fat ranged from 13%—31%, higher than in other intestinal segments [12].

However, these previous studies mainly focus simply on microbiome itself, while the interaction between host and microbiome can also contribute to the host phenotypes, such as fat deposition [13]. From ontogeny to homeostasis, complex organismal phenotypes are shaped by bidirectional interactions between host organisms and their associated microbiota. Although genomic and metagenomic studies have been instrumental in understanding many biological processes, each type of study has tended overlook the impact of the other, particularly the interactions between them. Recently, the recognition of the importance of these host-microbiota interactions has opened new avenues of research based on the integrated analysis of coupled genomic and metagenomic data [14]. Therefore, it will improve our understanding of genetic mechanisms of complex traits by taking microbiome and host as a whole, i.e., from the viewpoint of holo-omics [5]. Statistical models play an important role in the design of competent breeding programmes associated with complex traits. Recently, the holo-omics concept has been used effectively for trait prediction, and it has proved desirable to build predictions with accuracy while combining genomic and microbial data from the host [15]. Xu believes that such holo-omics studies have the ability to resolve the function of plant-microbiota ecosystems by generating images of expression, translation and production during plant–microbe interactions. And it was mentioned that the most commonly included type of host data currently available is transcriptomics, which provides a broad and in many cases relatively well-annotated view of host function [16].

Meanwhile, in order to obtain more findings, cross-breeds comparison of gut microbiota was regularly used in previous studies [11], but it is difficult to exclude confounding influence caused by breed differences. Therefore, in this study, individuals with extreme high or low obesity indicators within the same breed were used to reveal the holo-omics differences between them.

Although research over the last decade has established a strong link between gut microbiota and fat deposition, there is still a need to explore the causal relationships and potential mechanisms from the view of holo-omics. In this study, we tried to explore the holo-omics mechanisms of fat deposition by carrying out comparison between Jinhua pigs with high and low obesity indicators, while taking into account the spatial heterogeneity of the gut, so as to provide insights into both improvement of meat quality in pigs and understanding for obesity in human medicine.

## Results

### Phenotype characterization of the high and low fatness Jinhua pigs

**Fig. 1:**
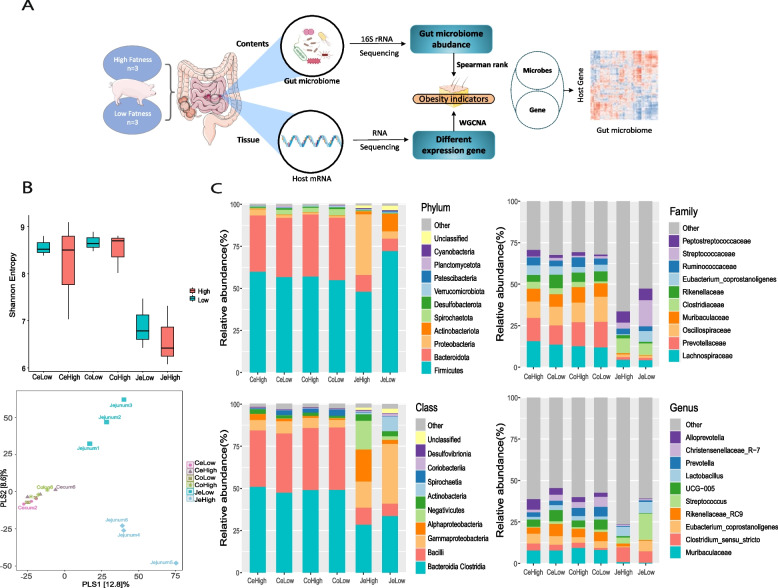
|Differences in microbial diversity and composition between low-fatness and high-fatness. **A** The experimental design of this study. **B** Comparison of the Shannon index in the three intestinal segment regions between the two groups. Mann-Whitney U test was performed to verify the differences, where *p* < 0.05 indicates significant difference. Partial least squares discriminant analysis of the composition of microbial species in the three intestinal segments. **C** Relative abundance of the top 10 microbial species at the phylum, class, family and genus level in the three intestinal regions of the two groups

In this study, we included 6 Jinhua pigs that were raised under standard management conditions. At 240 days of age, the pigs were slaughtered to measure body weight, backfat thickness, and high-density lipoprotein cholesterol (HDL-C) levels in the blood serum (Fig. 1A). Backfat thickness was identified as the most direct and objective indicator reflecting fat deposition in pigs. To support this finding, we also examined blood parameters related to obesity, such as triglycerides (TG) and high-density lipoprotein (HDL) levels. The high-fat group exhibited higher levels of these indicators compared to the low-fat group. The individuals were then divided into high-fat and low-fat groups based on the indicators of fat deposition (BF, HDL-C), as outlined in Table 1. Additionally, we measured phenotypic traits associated with meat quality, including eye muscle area and drip loss (Supplementary Table 1). To assess the significance of the differences in fatness phenotypes between the two groups, a t-test was performed on the relevant phenotypic indicators. The high-fatness group displayed significant differences from the low-fatness group in all phenotypes (*p* < 0.05), except for triglyceride and cholesterol lipid levels. Regarding meat quality traits, drip loss exhibited a significant difference between the two groups (*p* < 0.05). Furthermore, we computed the power value for these phenotype indicators, and the power value for the aforementioned indicators showing significant differences were all above 0.8 (Supplementary Table 2).

**Table 1:** **|** The comparison of phenotypes between the low-fatness and high-fatness groups

Phenotype	Low fatness (*n* = 3)	Fat fatness(*n* = 3)	*P*value
BW/kg	**79.067 ± 1.482**^**b**^	**83.667 ± 0.805**^**a**^	**0.029**
BF/cm	**5.909 ± 0.160**^**b**^	**6.467 ± 0.205**^**a**^	**0.042**
TG (mmol/L)	**0.797 ± 0.154**	**0.983 ± 0.199**	**0.358**
TC(mmol/L)	**3.037 ± 0.433**	**2.720 ± 0.205**	**0.422**
HDL-C(mmol/L)	**1.063 ± 0.034**^**a**^	**0.710 ± 0.008**^**b**^	**0.003**
LDL-C(mmol/L)	**1.823 ± 0.286**	**1.737 ± 0.115**	**0.721**
HDL-C/LDL-C (mmol/L)	**0.597 ± 0.090**	**0.410 ± 0.029**	**0.085**

Values in table are described with mean ± standard error

*BW* body weight, *BF* back fat, *TG* triglycerides, *TC* cholesterol, *HDL-C* high-density lipoprotein cholesterol, *LDL-C* low-density lipoprotein cholesterol

^a,b^Superscript letters in the same row mean a significant difference between the two groups (*p* < 0.05)

### The gut microbiome composition analysis of the high- and low-fatness pigs

Fecal samples form jejunum, cecum and colon were sequenced using 16S rRNA sequencing and clustered at 100% similarity to obtain characteristic sequence ASVs (amplicon sequence variants) for each gut segment microbiota. Supplementary Fig. 1 shows the number of ASVs obtained from each group and each intestinal region, as well as the ASVs that are co-occurring in each of corresponding samples. Overall, the large intestine region (Cecum and Colon) had more ASVs in the low-fatness group than those in the high-fatness group, whereas the reverse trend can be observed for small intestine region. However, there was no statistically significant difference in the number of ASVs between the groups (*p* > 0.05).

The Alpha diversity of each group was compared to illustrate the bacterial diversity of each sample (Fig. 1B). By comparing the Shannon index, the jejunum region was significantly less diverse compared with the cecum and colon regions (*p* < 0.05). The space and environment in large intestine are more suitable for microbes than the small intestine, resulting in an order of magnitude difference in the species and number of microorganisms in the two regions. In the comparison of the alpha diversity between the two groups in each region, the alpha diversity in high-fatness group was higher than that in the low-fatness group in the colon region. There was no major difference in the cecum region, while the Shannon index was lower in the high-fatness group than the low-fatness group in the jejunum region. None of the above results were statistically different. Such tendency can also be observed for other alpha diversity indices (Chao1, Simpson) (Supplementary Table 3). This suggests that the microbial composition in small intestine region of the high-fatness group is simpler than that of the low-fatness group, which is consistent with the results of previous studies on the microbiological composition of obese people [6].

Partial least square (PLS) analysis based on Bray–Curtis distance between pairwise of samples at the species level (Fig. 1B) shows that samples from jejunal region can be clearly divided across the second PLS components. In contrast, samples from cecum and colon regions are clustered together, while the first PLS component can clearly separate the jejunal samples from other samples in colon and cecum.

The relative abundance of dominant species in the three intestinal segments of the two groups was shown in Fig. 1C. Subsequently, the relative abundance of the microorganisms used for comparison was greater than 0.1% of the microbial population in the three intestinal regions of the two groups. These results indicate that jejunum and cecum have different microbial characteristics at the family and genus levels (Fig. 1C). According to the species classification results, *Bacteroidia* and *Clostridia* were the dominant organisms at the class level in all three intestinal segments, followed by *Bacilli* and *Spirochaetota* in the large intestine region. The proportion of *Alphaproteobacteria* and *Actinobacteria* in the small intestinal region determined the significant difference between the two groups (Fig. 1C). As the F/B(*Firmicutes*/ *Bacteroides*) is a candidate indicator for obesity, we also examined the F/B, which was decreased in jejunum of high-fatness pigs. However, there was no significant difference in the cecum and colon sections [17] (Supplementary Fig. 1).

### Analysis of microbial species differences

Meanwhile, to investigate the differences in microbial community abundance between the two groups. The LEfSe (Linear Discriminant Analysis Effect Size) method was used to find statistically different Biomarkers between the two groups (Supplementary Table 4).

**Fig. 2:**
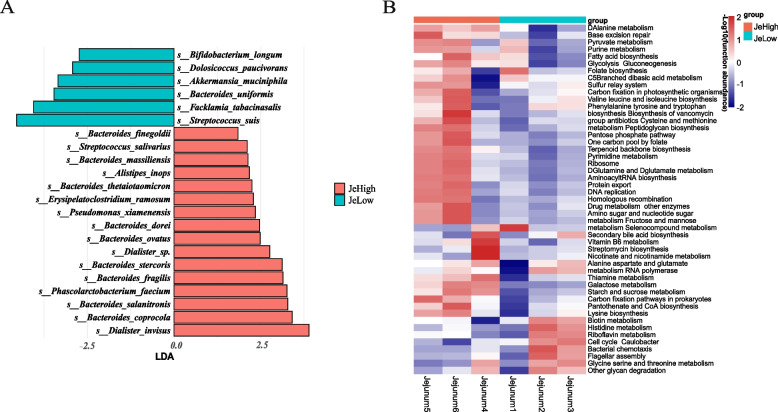
|Species abundance differences between high-fatness and low-fatness groups. **A** Search for marker microorganisms in the jejunal region using the LEfSe score discrimination. *q* < 0.05, LDA > 2 as the criterion for significant difference between the two groups. **B** The microbial constituent genes of the three intestinal segments were functionally enriched and an attempt was made to cluster them according to grouping and functional abundance

At the species level, a total of 22 different species from the two groups were identified in the jejunum region by the above LEfSe (Fig. 2A). There are 10 species belonging to the genus *Bacteroides*, including *Bacteroides ovatus*, *Bacteroides dorei* and *Bacteroides uniformisd*. In the cecum region, there are significant differences between the three microbial species. The abundance of *Eubacterium siraeum* and *Treponema porcinum* in the low fatness group is higher than that in the high fatness group, while *Clostridiales bacteriumc* presents the opposite trend. In the colon region, it is worth noting that in the high fatness group, the abundance of *Bacteroides plebeius* increased significantly. *Bacteroides plebeius* seems to be related to the environment and eating habits. Studies have found that the bacteria have significant differences in the two groups of Koreans living in the United States and South Korea. The two groups of Koreans have very different geography and eating habits, and the bacteria is significantly abundant in the high-calorie diet environment in the United States [18]. Also from the side, the bacteria will exist in individuals with high energy storage and metabolism.

In order to further understand the function of these species in the host, we performed functional enrichment for the microbial composition genes of the three intestinal segments, and attempted to cluster according to grouping and functional abundance. The results showed that the cecum and colonic segments had obvious similarity in function, and the clustering was not obvious (Supplementary Fig. 2). However, the two groups in the jejunum region could be obviously clustered apart. There were significant differences in enrichment of "Fatty acid biosynthesis", "Glycolysis Gluconeogenesis" and "Peptidoglycan biosynthesis" (Fig. 2B).

From the functional annotation of the differential bacteria species between the two groups (Fig. 2B), we suggested the pathways by which the microbiology cause elevated obesity indicators in pigs may be the following: 1) Associated species can cause chronic inflammation of the intestinal tract, leading to fat accumulation in the host [19]; 3) The metabolites of the species in question can promote or inhibit the production of adipocytes, consequently affecting on host phenotype [20].

### The intestinal transcriptome landscapes

**Fig. 3:**
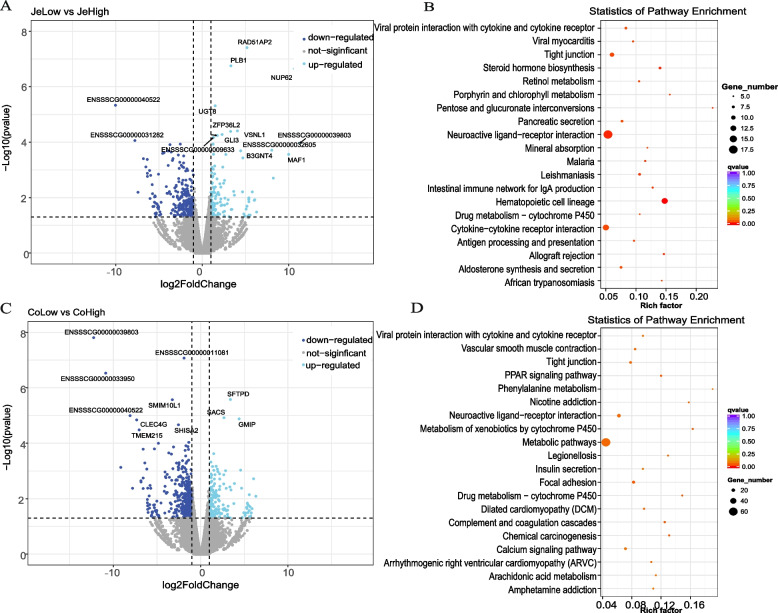
|Results of DEGs of low-fatness and high-fatness -in jejunal and colonic tissues. **A**, **C** The two groups of differentially expressed gene profiles including the number of upregulated and downregulated genes in the jejunum and colon regions are shown by volcano plots. Down-regulated: *p* < 0.05 & log2foldchange < -1; Up-regulated: *p* < 0.05 & log2foldchange > 1. **B**, **D **KEGG pathway enrichment analysis of differentially expressed genes between the two groups. y-axis shows the name of the pathway and x-axis shows the enrichment factor. Pathways with significant enrichment are shown in the KEGG scatter plot. The enrichment factor is the ratio of the number of differentially expressed genes to the number of all genes annotated in a particular pathway. The q-value, obtained through the Benjamini-Hochberg (BH) method, is a corrected value for the p-value. A q-value less than 0.05 is considered statistically significant, indicating significant findings after controlling for multiple testing

It is important to clarify that the results showed that the cecum had the highest number of differential genes (Supplementary Table 6) of all three intestinal regions. However, during the mapping step, it was found that one of the samples from the cecum region was only mapped to about 20% of the reference genome due to microbial contamination. Therefore, in our transcriptome analysis, we only focused on the jejunal and colonic regions to ensure the reliability of the results. In the jejunal region, 126 genes were up-regulated and 258 genes were down-regulated in the low-fatness group relative to the high-fatness group (Fig. 3A). Functional enrichment results show that jejunum have significant differences in some receptor actions: "Cytokine-cytokine-receptor interaction", "Hematopoietic cell lineage", "Neuroactive ligand-receptor interaction" (Fig. 3B). In the colonic region, 594 genes were screened for differences, of which 175 were up-regulated and 419 were down-regulated in the low-fatness group compared to the high-fatness group (Fig. 3C). Notably, there were significant changes in lipid metabolism-related pathways between the two groups, including "Pancreatic secretion", "Steroid hormone biosynthesis", and "Arachidonic acid metabolism", which were also enriched in the jejunum (Fig. 3D). The most prominent pathway enriched in the colon was the “Metabolite pathway”. The differences between the two groups may be due to the action of certain active substances in the metabolic pathways, which affect lipid metabolism and result in different phenotypes. These important 'communication substances' are likely to be metabolites of microorganisms that are enriched in the gut. Finding the link between the two and bridging the host-microbe interactions will be the next step.

In order to investigate which intestinal region has larger influence on lipid production, we identified DEGs between colonic regions (large intestine) and jejunal regions (small intestine) for low and high fatness groups, respectively (Supplementary Fig. 3). The pathways involved in lipid metabolism were significantly enriched, suggesting that the two sites are significantly different in their roles in "Fat digestion and absorption", "Cholesterol metabolism", "Arachidonic acid metabolism" and "Insulin resistance".

LncRNAs can help detect distinct functional pathways resulting from various therapies and affect the transcriptional and post-transcriptional levels of target gene expression [21]. After mapping to the reference genome, transcript splicing was identified. In this step, we also carried out transcript screening, using Cuffcompare software to compare with known databases, filtering out known transcripts from databases, and finally obtaining 18,341 novel lncRNAs (Supplementary Table 7). The novel lncRNAs were classified into 3 main types: antisense, lincRNA, and sense overlapping, based on their position in relation to known mRNAs, with reference to HGNC [22].

**Fig. 4:**
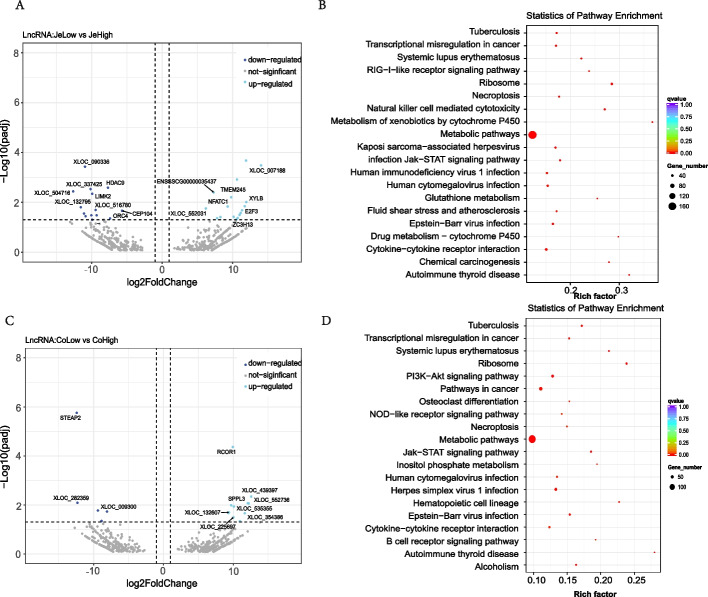
|Prediction and functional analysis of LncRNA. **A**, **B** Functional enrichment of genes in LncRNA localized regions in jejunum and colon. Down-regulated: *p* < 0.05 & log2foldchange < -1; Up-regulated: *p* < 0.05 & log2foldchange > 1. **C**, **D** KEGG pathway enrichment analysis of differentially expressed genes between the two groups in jejunum and colon. The q-value, obtained through the Benjamini-Hochberg (BH) method, is a corrected value for the p-value. A q-value less than 0.05 is considered statistically significant, indicating significant findings after controlling for multiple testing

Regulation of target genes by lncRNAs occurs through a variety of approaches, we focus here on the case of target genes within 100 kb upstream and downstream of the lncRNA for functional enrichment analysis. The results showed that genes near the differential lncRNAs for jejunal and colonic intestinal segments were mostly enriched in the “metabolic pathway”. Furthermore, the enrichment analysis of lncRNA target genes showed that two intestinal regions were enriched in more pathways related to lipid metabolism: ''fatty acid metabolism'', ''biosynthesis of unsaturated fatty acids'', and ''regulation of lipolysis in adipocytes''. This is consistent with the results of the previous mRNA studies (Fig. 4).

We also predicted circRNAs (Supplementary Table 8) and identified differentially expressed ones which were further used to obtain enriched KEGG functional pathways (Supplementary Fig. 4). The results showed that in addition to the conventional metabolic pathway, the mTOR signaling pathway was also enriched. mTOR signaling pathway has been shown to influence fat deposition through metabolite regulation in the body. The miR-181 family, which is highly bound to the identified target genes, was also found to be regulated by metabolites for brown fat deposition in the intestine [23].

### Associations of microbe–intestine interactions with obesity indicators

To further explore the interactions between the host and gut microbiomes and to validate the above studies, we used a multi-omics approach to correlate obesity indicators, gut transcriptome and microbial species abundance. Briefly, the WGCNA R package was used to identify key gut tissue genes within each gut region that were significantly associated with indicators of obesity. After the analysis, a total of 93 key genes were identified in the jejunum region, while 43 key genes were identified in the colon region (Supplementary Table 9). These key genes can now be subjected to further analysis for subsequent investigations. In the previous analysis, we obtained two sets of differentially abundant microbial species from different gut regions. After filtering based on abundance, we selected 73 microbial species from the jejunum region and 24 microbial species from the colon region for correlation analysis with key genes using the Spearman method (Supplementary Table 10).

**Fig. 5:**
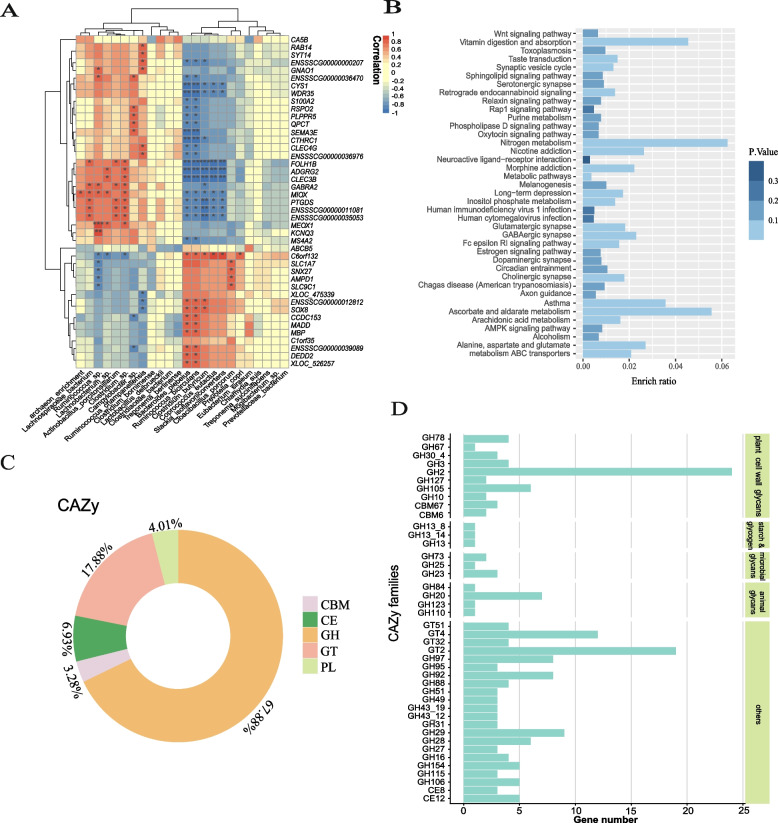
|Associations of Microbe–Intestine Interactions With Obesity indicators. **A** We correlated the key genes associated with fat deposition with differentially abundant microbiota and calculated the correlation coefficient (Spearman's coefficient). The heatmap's color indicates the degree of correlation.*:*p* < 0.05, **:*p* < 0.01, ***:*p* < 0.001 **B** Differential genes associated to Bacteroides plebeius species with significant differences in the colonic region were enriched for KEGG pathways of high relevance to them. Functional entries were found to focus on intercellular communication functions. **C** Types and proportions of carbohydrases encoded by the Bacteroides plebeius genome. **D** The functions of the enzymes of interest were investigated. The results showed that the bacterium had the highest number of genes encoding GH2, GH20, GT4 and GT2

The results showed that the gene modules in each region were mainly associated with body weight (BW) and backfat thickness (BF) phenotypic indicators. In the jejunal region, the proportion of gene modules associated with blood obesity indicators (HDL, TC) was higher than in the large intestine region, we presented them in the form of heat maps and clustered the module genes (Supplementary Fig. 5). In the colon, two microbial species(*Bacteroides plebeius, Clostridium butyricum*) that both increased in the high-fatness group (Fig. 5A). We utilized multivariable regression analysis, with gene expression levels as the dependent variable and microbiota as the independent variable, to construct a linear regression model. we estimated the coefficients of the independent variables and assessed their significance. *Bacteroides plebeius*, *Clostridium butyricum* again showed significant associations with key genes, and in the same direction as the spearman correlation described above (Supplementary Table 11). Then, we focus on the genes that are associated with significant variations in two microbial species and examine their functions. The results of the functional enrichment revealed three main categories involved, the first involving the metabolism of vitamins and amino acids, with particular emphasis on glutamate metabolism. The second category includes a range of intercellular communication pathways, while the third is mainly concerned with lipid metabolism, for example the metabolism of arachidonic acid. In the colon tissue, *FOLH1B*, which is significantly associated with microbes, encodes a receptor for intestinal folate. It can indirectly regulate microbial metabolic capabilities [24]. In the genes related to obesity indicators, 93 jejunal DEGs expression levels were found to be significantly correlated with microbial species (*p* < 0.05). Notably, *APOH* is involved in cholesterol metabolism pathways. The cecum fraction was not analyzed for association as the cecum tissue samples received contamination.

In the above results we noticed that *Bacteroides plebeius* not only differed significantly between the two groups in the colon region, correlating with indicators of fat deposition, but that its significantly associated gene function enrichment results also showed an association with fat metabolism (Fig. 5B). Therefore, we explored further the coding function of this bacterium. Immediately afterwards, the ability of this species to encode carbohydrates was also explored. A total of five types of carbohydrates were identified, of which "GH (Glycoside Hydrolases)" and "GT (Glycosyl Transferases)" were the main components (Fig. 5C). The functions of the enzymes of interest were also investigated. The results showed that the bacterium had the highest number of genes encoding GH2, GH20, GT4 and GT2 (Fig. 5D). This suggests that the strain is likely to be involved in lipid metabolism and the regulation of cellular communication in the intestine through different pathways, while at the same time suggesting that the microbial influence on the host is widespread. This partly explains why these genera show correlations with fatty acid binding proteins in terms of developmental changes and fat deposition.

## Discussion

The gut is now widely recognized as an influential factor affecting growth and development as well as performance indicators in pigs. And these studies have described functional changes in the composition and gut microbiome of obese individuals, which reveal a strong correlation between the gut microbiota and obesity. Nowadays, numerous studies investigating the structure of the gut microbiota have been proposed to reveal the relationship between gut bacterial species and pig obesity through metagenomics and 16 s in pigs. We explored the interactions between two groups with differences in obesity phenotypes, including differences in gut microbiota, and gut tissue expression, within the Jinhua pig population. In addition, we sought to explore the mechanisms by which intestinal genes and microbes interact to cause fat deposition.

Previous studies have found that high oxidative stress and dysregulated ecology of lipid metabolism in high-fatness pigs may be responsible for fat deposition in gilts. Host fat deposition is affected through the methanogenic function of archaea and the production of short-chain fatty acids by bacteria [25]. Developmental changes in the structure and expression levels of fatty acid binding proteins in the ileal flora of Jinhua pigs, and correlation with fat deposition [26]. The intestinal fungal structure of Jinhua pigs with different fatness rates differed and correlated with backfat thickness, indicating a correlation between intestinal fungal changes and host fat deposition [27].

HDL is a protein that plays an important role in the body and has an anti-atherosclerotic effect. Reduced HDL levels are often associated with obesity, hypertension, dyslipidaemia and insulin resistance, and the body can develop metabolic disorders [28]. Lipocalin concentrations have been found to correlate with lipoprotein metabolism, particularly with the metabolism of HDL and triglycerides [29]. Studies have found a significant negative correlation between HDL cholesterol and coronary heart disease, meaning that if HDL is reduced, the risk of cardiovascular disease and atherosclerosis increases. Therefore, in this study we used HDL also as an indicator to determine the obesity phenotype of Jinhua pigs.

In terms of the number of species, high-fatness is relatively homogeneous with fewer numbers and species compared to low-fatness. This is consistent with previous studies that have shown the relatively simple flora structure of obese individuals [6, 30]. We hypothesize that the occurrence of microbially induced fat deposition in the body may have a strong correlation with the absorption and metabolism of the small intestinal fraction, especially for fat metabolism and absorption. The functional prediction of the bacterial community suggested increased fatty acid biosynthesis in Jinhua pigs, which could partially explain their adiposity phenotype [11].

The differential genes in the large intestinal segment were invariably able to enrich for the metabolic pathway in question, further validating our suspicions. The differential cricRNA enrichment pathway to the mTOR signaling pathway, which can be regulated by the intestinal metabolite imipramine, activates p38-p62-mTORc1, which in turn inhibits the function of insulin receptor substrates, blocking the insulin receptor pathway and leading to insulin resistance. In turn, insulin resistance leads to lower HDL, higher serum triglycerides and higher LDL [23].

RNA binding motifs, potential transmembrane structural domains and proline-rich regions were significantly correlated with body weight in jejunal and colon tissues. In colon tissue CLEC4G again showed a significant correlation with backfat thickness. This gene encodes a glycan-binding receptor and a member of the C-type lectin family that functions in the immune response. C-type lectin receptors are pattern recognition receptors located on immune cells and play a role in the recognition and uptake of self and non-self glycoproteins and in mediating cell adhesion, glycoprotein clearance and cell signaling functions.

We therefore have the following speculations on the mechanism of action of microbial-organism interactions causing obesity, 1) Immune-related differentially expressed genes interact with metabolites produced by bacteria to drive anti-inflammatory responses to prevent obesity and 2) Key species are likely to be involved in lipid metabolism in the gut as well as in regulating cellular communication through different pathways that allow developmental changes in fatty acid binding proteins. However, our study still leaves much to be desired, whether the small sample size investigation represents a universal pattern remains to be further validated. Additionally, the lack of recorded body size data limits our ability to account for inherent variability in body size measurements. And secondly many genes with no clear function were also identified as microbial interactions associated with fat deposition in Jinhua pigs, suggesting an integrated interaction between the gut microbiota and host genes, which needs to be fully described by more studies.

## Conclusion

In summary, differences in gut microbes structure as well as abundance have a differential impact on the fat metabolism of the host. The metabolic pathways of substances in the digestive tract are disturbed, which leads to differences in gene expression in the intestinal tissues, ultimately affecting the digestion and absorption of key substances or the delivery of small molecules, cascading down to an obese phenotype.

This study examines how the gut microbiome affects obesity indicators in pigs in terms of gut tissue development, providing hints for improving growth performance and fat deposition levels in local pigs.

## Methods

### Animal experiments and sample collection

This study aimed to investigate the relationship between the fatness of pigs and their gut microbiota composition. The researchers fed 18 newly born Jinhua pigs a standard corn-soybean-based diet for 240 days and then collected blood samples, intestinal tissue, and abdominal adipose tissue from each pig. The pigs were sourced from the same environment (pig farm), received the same feed, were of the same age (240 days), and of the same gender (female), ensuring minimal differences in the designed traits. The specific composition of the feed, provided by Farm for the two groups of Jinhua pigs, was consistent with the regular feed used in their daily feeding. Upon slaughter, the pigs are immediately exsanguinated, and blood is promptly collected and transferred into tubes. Centrifugation was performed using a centrifuge at 4° C until the blood was stratified and the upper serum was extracted. The tubes are then placed on ice or in a cold pack at temperatures ranging from 0℃—4 ℃ to maintain the integrity of the blood sample. Following the removal of hair, the heads, hooves, tail, and internal organs are discarded, while the body weight is measured by preserving the fat layer and kidneys, which represent the body weight (BW). The collected blood samples, stored at 0℃—4 ℃, are transported back to the laboratory at Zhejiang University for analysis. The time between sampling and testing does not exceed 6 h to ensure the stability and reliability of the samples. All experimental procedures adhere to the aforementioned requirements for blood parameter analysis. To assess fat deposition traits, we considered both phenotypic measurements and blood parameters. Among the various phenotypic measurements reflecting fat deposition in pigs, backfat thickness emerged as the most direct and objective indicator. In order to support this result, we also examined blood parameters. Key obesity indicators such as triglycerides (TG) and high-density lipoprotein (HDL) showed higher levels in the high-fat group compared to the low-fat group. The most extreme pigs (three high-fat and three low-fat pigs) were identified for further analysis using t tests for indicators of fat deposition. To further examine whether the sample size has statistical power, we performed Two-Sample t tests using the SAS software [31] to calculate the power values for each phenotype indicator when the total sample size was 6.

Luminal samples were collected from the same locations in the jejunum, cecum, and colon of the selected pigs, and 16S sequencing was performed on the intestinal contents. Additionally, 3 cm of intestinal tissue was collected from each pig and snap-frozen in liquid nitrogen for mRNA sequencing. The luminal samples were collected within 30 min after slaughter and stored in a -80 °C refrigerator until DNA extraction. It should be noted that all animal experiments were approved by the Zhejiang University Institutional Animal Care, and were conducted in accordance with relevant rules and regulations.

### 16S Ribosomal RNA gene sequencing

The gut microbial DNA was isolated from the three intestinal contents per pig using the QIAamp DNA Stool Mini Kit (Qiagen, Hilden, Germany) following the standard manufacturer’s protocol at benagen technology Institute (Wuhan, China).

Following the manufacturer's instructions, microbial DNA was isolated from three intestinal contents using a DNA extraction kit. The V3-V4 region of the microbiota 16S ribosomal RNA genes was amplified by polymerase chain reaction (95℃ for 3 min, followed by 30 cycles at 98℃ for 20 s, 58℃ for 15 s, and 72 ℃ for 20 s and a final extension at 72 ℃ for 5 min) using primers 341F 5’-CTACGGGRSGCAGCAG)-3’ and 806R 5’-GGACTACVVGGGTATCTAATC-3’. A 30 µl mixture including 15 µl of 2 × KAPA Library Amplification Ready Mix, 1 µl of each primer (10 M), 50 ng of template DNA, and double-distilled water was used to conduct the polymerase chain reaction experiment. According to the manufacturer's recommendations, amplicons were extracted from 2% agarose gels, purified using the AxyPrep DNA Gel Extraction Kit from Axygen Biosciences in Union City, California, and quantified using Qubit 2.0 (Invitrogen, United States).After preparation of the library, these tags were sequenced on the HiSeq platform (Illumina, Inc.,CA, United States) for paired-end reads of 250 bp, which were overlapped on their ends for concatenation into original longer tags. DNA extraction, library construction, and sequencing were conducted at benagen technology Institute (Wuhan, China).

Single sample sequence data were obtained by barcode identification of the mixed sample library; primer sequences were identified and removed using cutadapt [32] (version 3.4) software to obtain Clean sequences that did not contain primers.

Then, quality filtering on the raw tags was performed under specific filtering conditions to obtain high quality clean tags, and the reads were compared with the species annotation database (Silva) (http://www.arb-silva.de/) according to QIIME2 [33] quality controlling process to obtain the final clean reads. Chimeric sequences were detected and removed using the UCHIME algorithm [34]. The DADA2 [35] method focuses on quality filtering, denoising, splicing (illumina data only) and chimera removal. Sequences are clustered at 100% similarity and each de-duplicated sequence produced after QC is called ASVs (amplicon sequence variants). Use the R package “phyloseq” to draw all samples flat at minimum ASV abundance and filter for low abundance ASV.

Classify-sklearn algorithm using QIIME2 [33]: for each ASVs feature sequence, using the default parameters in the QIIME2 software. Species annotation was performed using a pretrained Naive Bayes classifier. At the same, using QIIME2 software, samples were evaluated for Alpha Diversity Index. Alpha diversity of observed ASVs, Chao1 index, Shannon index, Goods coverage, phylogenetic diversity and beta diversity. To ensure the accuracy of the results, we further processed the ASV abundance table, where 0 values were treated as missing values and only remained larger than microorganisms in 50% of the samples. To investigate the differences in microbial community abundance between the two groups of samples, Metagenomic biomarker search is achieved using LEfSe [36]. In this study, we consider *q* < 0.05, LDA > 2 are microorganisms with significant differences between the two groups, and the specific results are shown in Supplementary Table 4. PICRUST2 [37] predicts sample function based on the sequence abundance of marker genes in the sample, The classification function abundance values in the corrected KEGG in the sample were output.

### Transcriptome sequencing and analysis

Using the TRizol reagent, the total RNA of the corresponding all tissues was prepared for mRNA sequencing. The RNA integrity and yield were assessed by the RNA Nano 6000 Assay Kit of the Bioanalyzer 2100 system (Agilent Technologies, Santa Clara, CA, United States) and the NanoPhotometer spectrophotometer (IMPLEN, Westlake Village, CA, United States). 3 μg of RNA per sample was used to create sequencing libraries using the NEBNext Ultra TM RNA Library Prep Kit for Illumina (NEB, Ipswich, MA, United States) in accordance with the manufacturer's instructions. Index numbers were added to identify each sample's sequences. Finally, the clustered libraries were sequenced on an Illumina HiSeq platform, and 150-bp paired-end reads were generated.

The raw data obtained by sequencing contains a small number of reads with sequencing junctions or of low sequencing quality. In order to ensure the quality and reliability of the data analysis, the raw data needs to be filtered with the following filtering criteria: 1) Removal of reads with a splice (adapter);2) Removal of reads with a proportion of N (N means base information cannot be determined) greater than 0.002;3) When the number of low quality bases contained in a single-ended read exceeds 50% of the proportion of the length of that read, this pair of paired reads needs to be removed. After raw data filtering, sequencing error rate checking and GC content distribution checking, clean reads were obtained for subsequent analysis.

The clean reads are compared to the reference genome or transcriptome using HISAT2 [38] and the results are output as a standard SAM file. The percentage of reads in the exonic, intronic and intergenic regions of the genome is then counted according to the results of the alignment. We used Stringtie [39] software to splice the reads into transcripts and quantify them based on the results compared to the genome. We then used Cuffmerge to merge the transcripts obtained by splicing each sample, removing those with uncertain strand orientation and transcripts up to 200 nt in length, then used Cuffcompare to compare with known databases, filtering out known transcripts, and finally performing coding potential prediction on the filtered new transcripts.

A comprehensive investigation of transcriptome, including coding and non-coding RNAs, was performed in the three intestinal regions of Jinhua pigs. Firstly, RNA was extracted from a total of 18 intestinal tissues from three intestinal regions of 6 pigs. After quality was determined, RNA sequencing was performed on these samples using Illumina HiSeq platform. A total of 1,606,004,430 raw reads with a length of 150 bp were retrieved from the sequencing of 18 libraries. After quality control, a total of 1,577,466,652 clean reads were remained for each sample and the proportion of Q30 bases was more than 90% (Supplementary Table 5).

The expression level of a gene is directly reflected in the abundance of its transcript. In order to make the estimated gene expression comparable across genes and different experimental conditions, we calculated FPKM (FragmentsPer Kilo bases per Millionreads) values for difference comparison. To address the issue of zeros in transcriptome data, we chose the method of zero exclusion, which completely excluded zero from the analysis, retaining only those samples that were greater than zero in the 50% median sample, and the remainder were excluded as missing values. Afterwards, we used the software edgeR [40] for differential expression analysis and clustered the expression values of the samples using a hierarchical clustering approach. *p* < 0.05 and |log2foldchange|> 1 were used as criteria for significance of differences. Specific results are presented in Supplementary Table 6. For gene functional enrichment analysis, KEGG enrichment was subsequently performed using the cluster Profiler R package (v3.12.0) and the pathway profiles in the KEGG database.

### Functional analysis of non-coding RNA

The target gene of the lncRNA is predicted by the positional relationship (co-location) and expression correlation (co-expression) of the lncRNA with the protein-coding gene. GO and KEGG enrichment analysis of target genes for co-location and co-expression of differential lncRNAs, respectively, to predict the function of lncRNAs.

In this project we used find_circ [41] and CIRI [42] to identify circRNAs, the two methods differ in principle and the combined analysis improves accuracy. The origin of circRNAs and the distribution of chromosomal positions were counted. The expression of known and new circRNAs in each sample was counted and normalized using TPM. Differential and enrichment analyses were performed on the results. The significance criteria were the same as those for mRNAs described above.

### Multi-omics association analysis

The phenotypes were compared statistically using R (4.1.3) for t-test for differences between high-fatness and low-fatness. We conducted a Weighted Gene Co-expression Network Analysis (WGCNA) [43] using the R package to assess the correlation between the obesity phenotype and mRNA gene expression. Our aim was to identify genes associated with adipose deposition in the jejunal and colonic regions. WGCNA clusters genes into multiple modules, and in this study, we specifically focused on selecting genes that exhibited significant correlation (*q* < 0.05) with the obesity phenotype indicators (Supplementary Table 9). These genes were chosen for subsequent omics-related investigations. The handling of zero values in the microbial abundance table is performed using the following steps: Firstly, zero values are treated as missing values, and only zero values that are present in less than 50% of the samples are retained for further analysis. Additionally, we add a small value of 0.01 to abundance data. Afterwards, we performed data transformation using the CLR (centered log-ratio) method on the selected differential microbial abundance matrix. We then conducted Spearman correlation analysis between the transformed microbial abundance matrix and the expression level matrix of the key genes obtained as mentioned above. We considered a correlation threshold of |r|> 0.6 and *p* < 0.05 as the criterion for identifying microbial-gene associations. The results of this analysis are presented in Supplementary Table 10. The results of microorganisms and associated genes in the jejunal region are shown in Supplementary Fig. 6. We utilized the "lme4" R package [44] to construct a linear regression model, with microbial abundance as the independent variable and key gene expression as the dependent variable. Through this model, we obtained the estimated effect values of each key gene on the microbiota, allowing us to assess the degree of association (Supplementary Table 11).

### Functional exploration of *Bacteroides plebeius*

We downloaded the assembled genome data of *Bacteroides plebeius* from NCBI [45]. The open reading frames (ORFs) in the genome were predicted using Prodigal software [46]. A comparable “faa” file was generated containing the protein sequences. Subsequently, the protein sequences were subjected to functional annotation using the kofam_scan software [47]. This annotation was based on Hidden Markov Models (HMMs) and the kofam database, enabling the identification of protein sequence homology and completion of gene function annotation. The type and number of carbohydrase encoded by the assembled genome were annotated using the CAZY database [48].

### Statistical analysis

*P* < 0.05 was considered statistically significant. In addition, a Benjamini–Hochberg false-discovery rate-corrected pvalue (qvalue) was estimated. The corrected results were included in the Supplementary Tables 4, 6, 9, and 10.

### Supplementary Information

**Additional file 1:** **Supplementary Table 1.** Phenotype. **Supplementary Table 2.** Two Sample Group t Test. **Supplementary Table 3.** Alpha diversity. **Supplementary Table 4.** LEfSe(Line Discriminant Analysis (LDA) Effect Size. **Supplementary Table 5.** The basic statistics of mRNA-seq data. **Supplementary Table 6.** high-fatness and low-fatness differentially expressed genes (mRNA). **Supplementary Table 7.** Novel LncRNA information. **Supplementary Table 8.** Novel-circRNA.  **Supplementary Table 9.** Key gut tissue genes within each gut region that were significantly associated with indicators of obesity. **Supplementary Table 10.** Correlations of phenotype-related microorganisms with key genes. **Supplementary Table 11.** Estimated effect sizes from microbial linear regression models

**Additional file 2.** 

**Additional file 3.** 

**Additional file 4.** 

**Additional file 5.** 

**Additional file 6.** 

**Additional file 7.**

## Data Availability

The sequencing raw data have been deposited into the Sequence Read Archive Database of National Center for Biotechnology Information (NCBI). The BioProject accession number is PRJNA1010517 (https://www.ncbi.nlm.nih.gov/sra/?term=PRJNA1010517).
