# Supplementary material for: Holo-omics analysis reveals the influence of gut microbiota on obesity indicators in Jinhua pigs
Source: BMC Microbiol. 2023 Nov 3;23:322. doi: 10.1186/s12866-023-03011-8 (PMC10623862; doi:10.1186/s12866-023-03011-8)

A Analysis statistics of the number of ASVs for each group

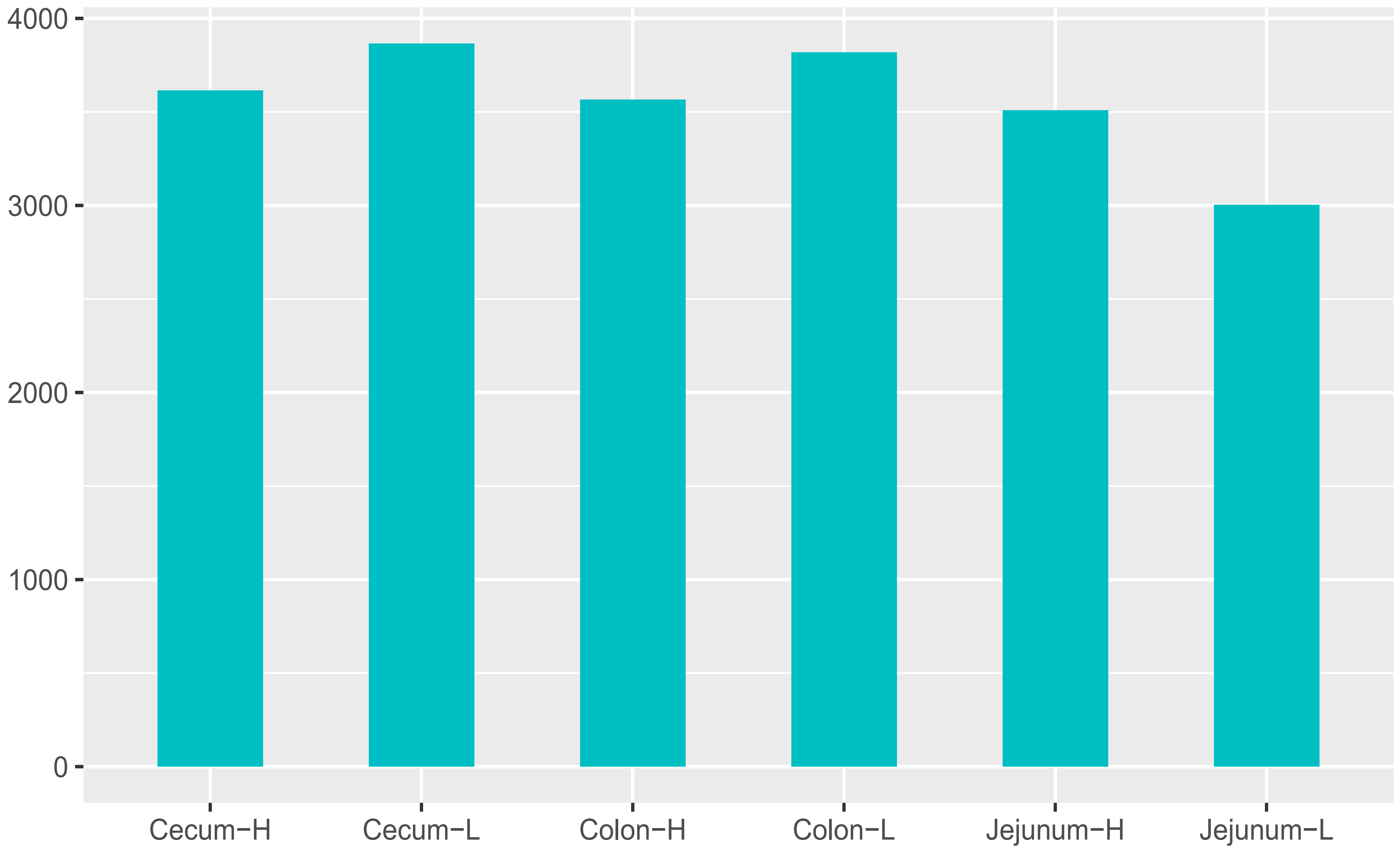

B F/B (Firmicutes / Bacteroides) ratios in the three regions

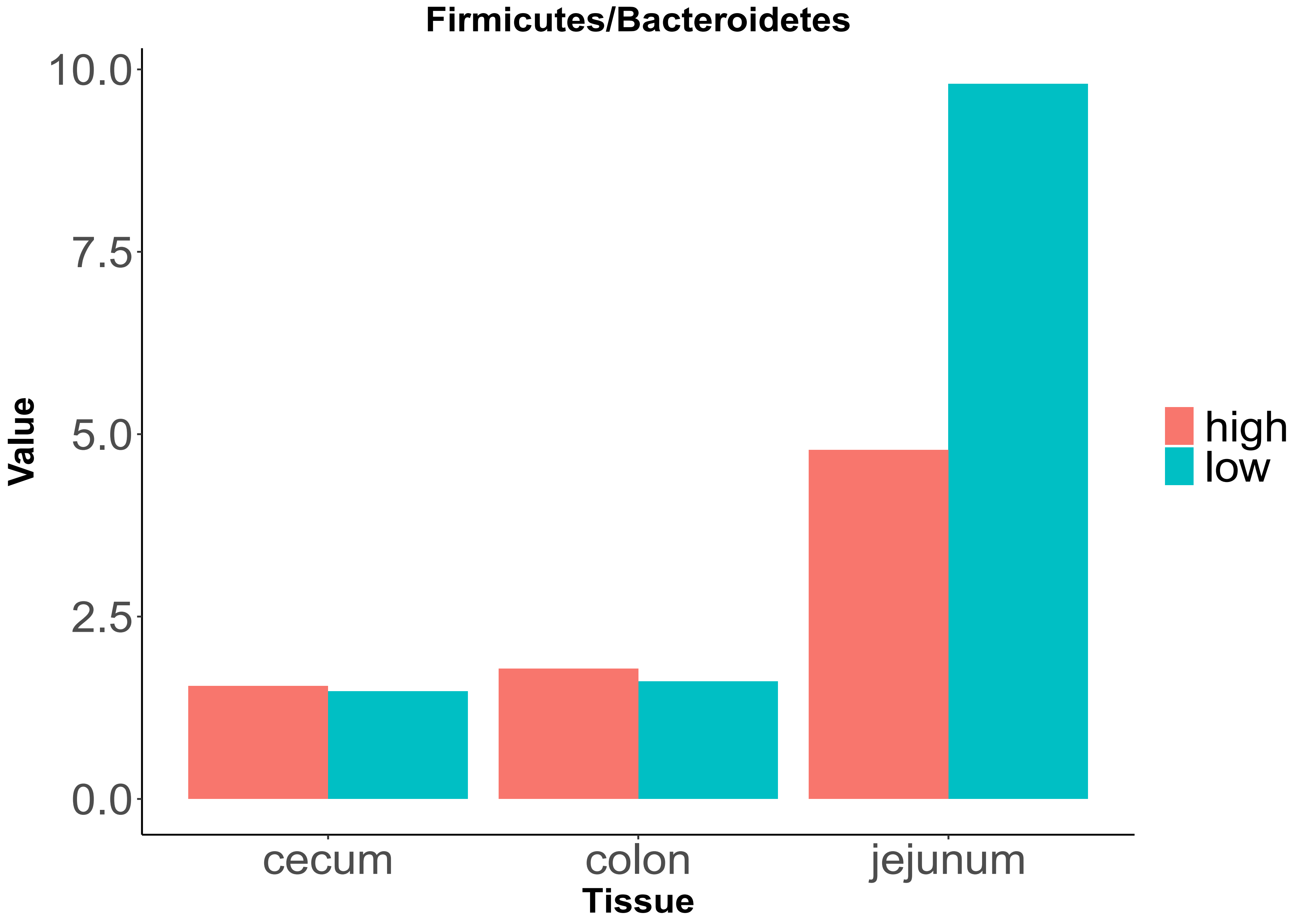

Supplement: Supplementary file 2 — Additional file 2. [file 12866_2023_3011_MOESM2_ESM.pdf]
