# Supplementary figures and images for: Holo-omics analysis reveals the influence of gut microbiota on obesity indicators in Jinhua pigs
Source: BMC Microbiol. 2023 Nov 3;23:322. doi: 10.1186/s12866-023-03011-8 (PMC10623862; doi:10.1186/s12866-023-03011-8)

Microbial functional enrichment in cecum and colon

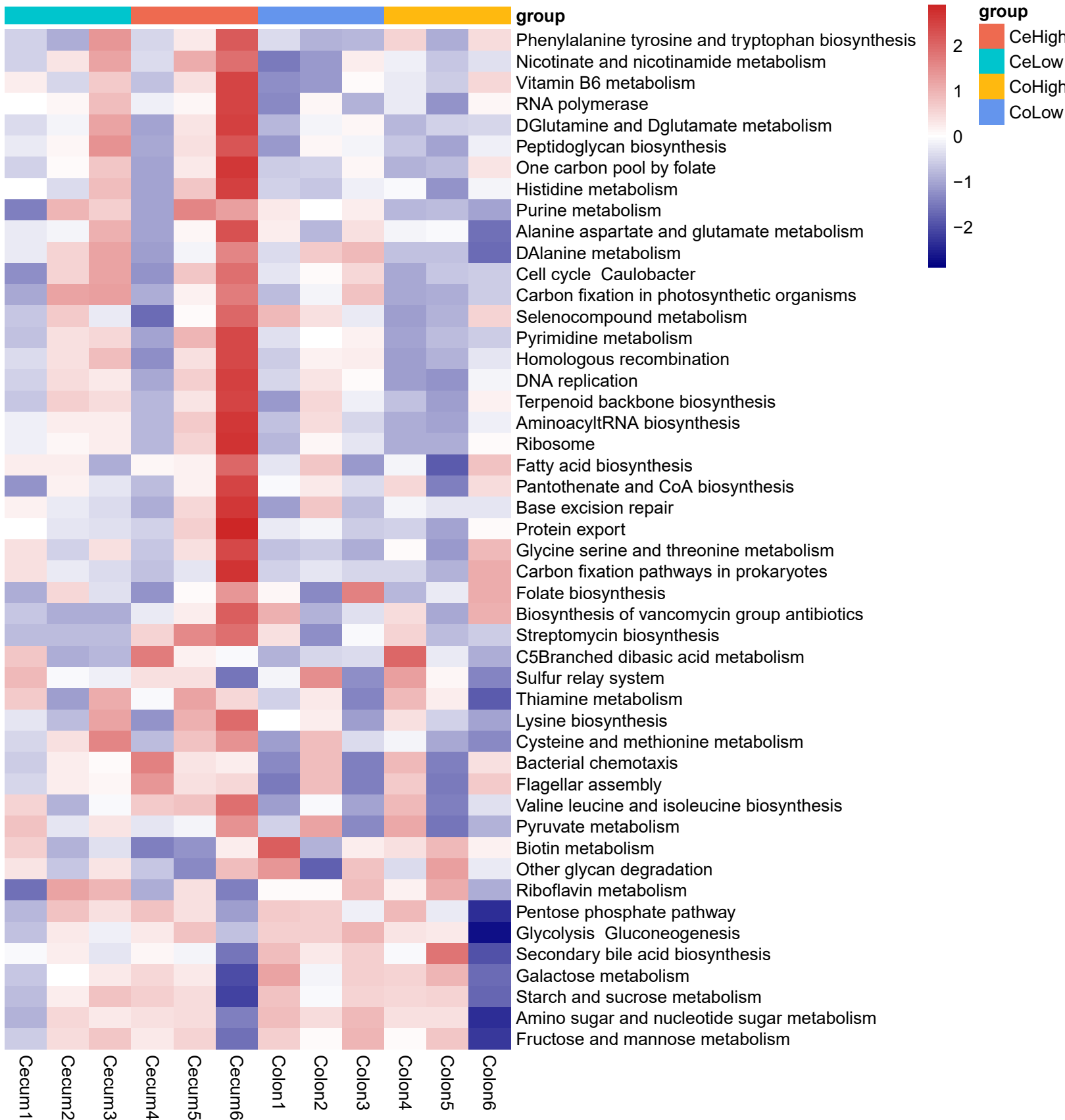

Supplement: Supplementary file 3 — Additional file 3. [file 12866_2023_3011_MOESM3_ESM.pdf]

Results of functional enrichment of predicted target genes

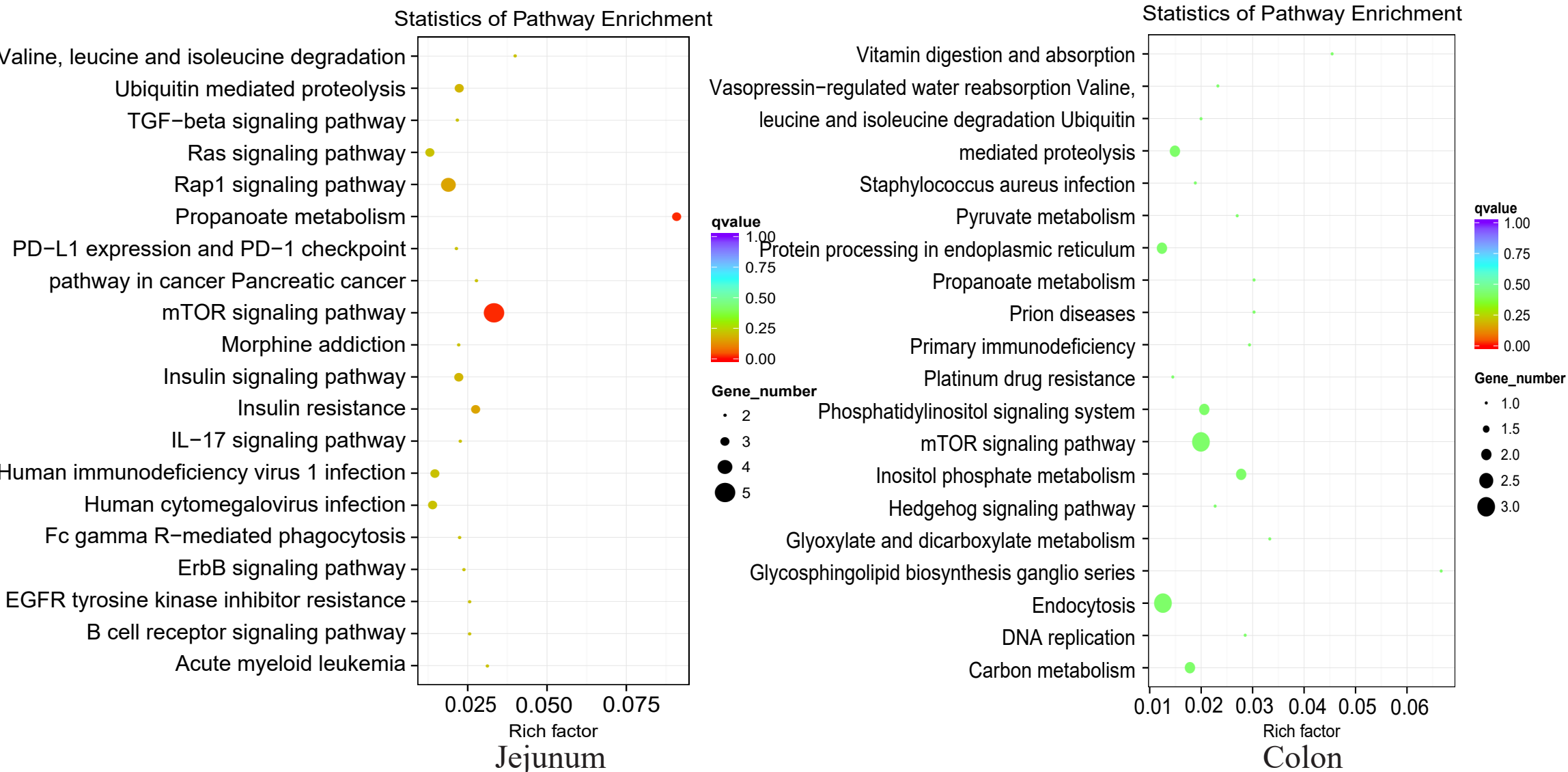

Supplement: Supplementary file 5 — Additional file 5. [file 12866_2023_3011_MOESM5_ESM.pdf]

WGCNA module plot

Cluster Dendrogram

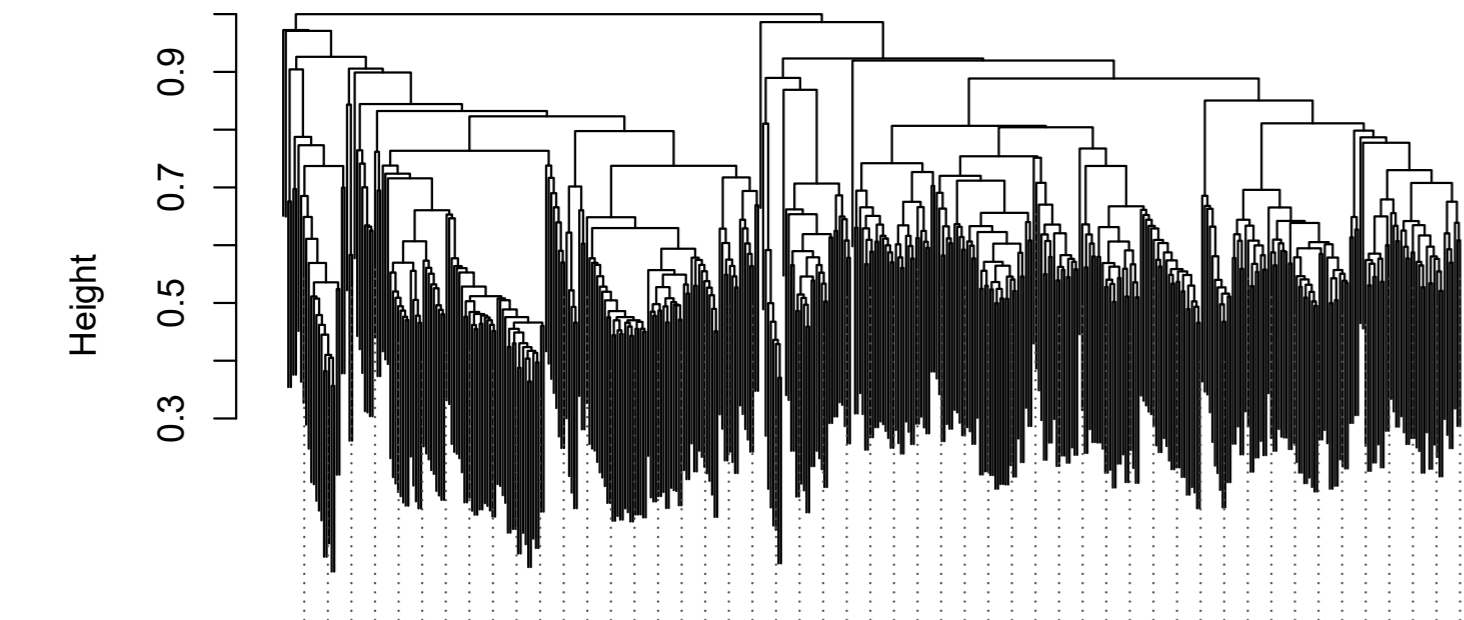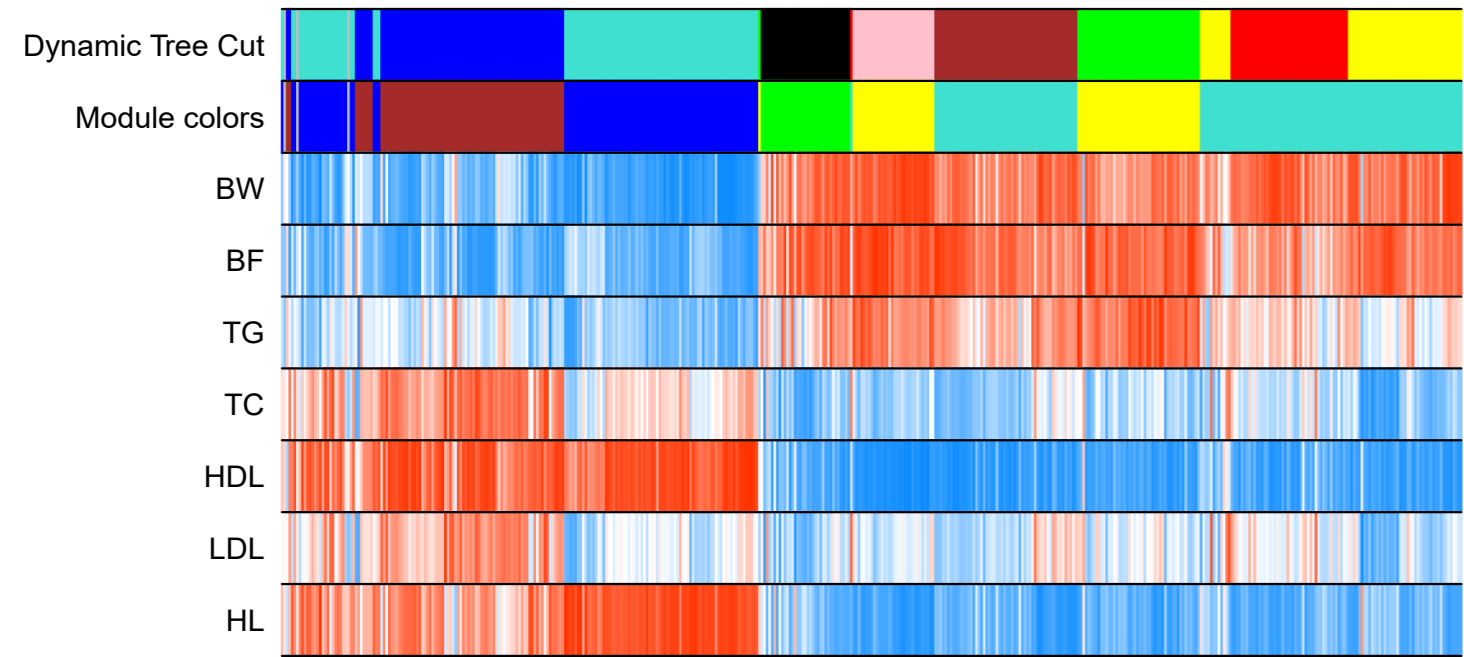

Jejunum

Cluster Dendrogram

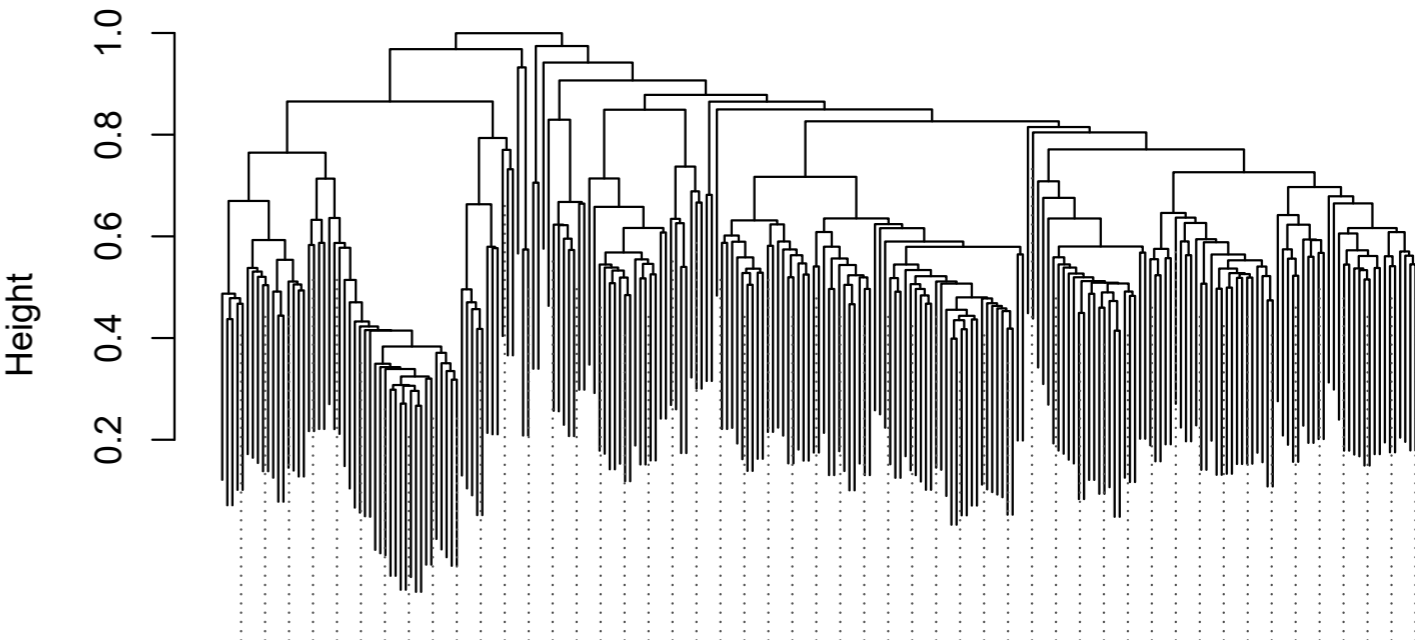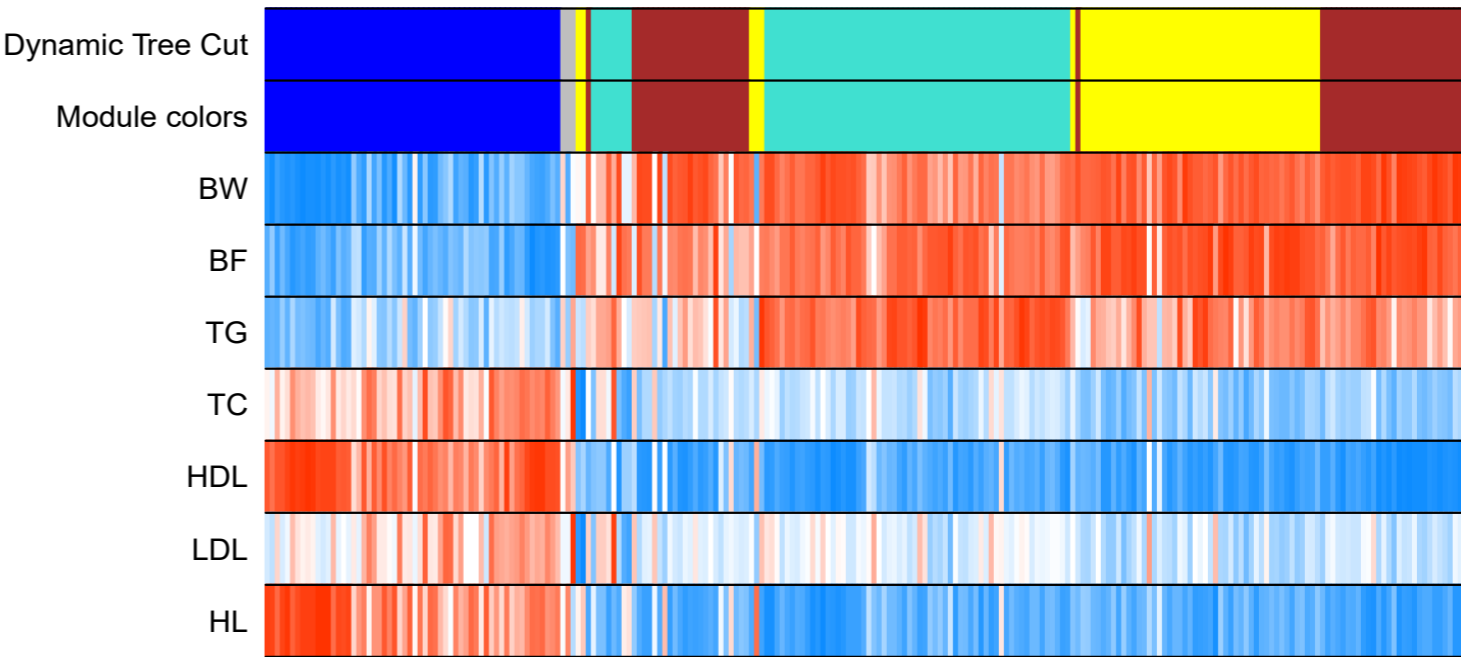

Colon

Supplement: Supplementary file 6 — Additional file 6. [file 12866_2023_3011_MOESM6_ESM.pdf]

# Association of microorganisms with key genes in the jejunum region

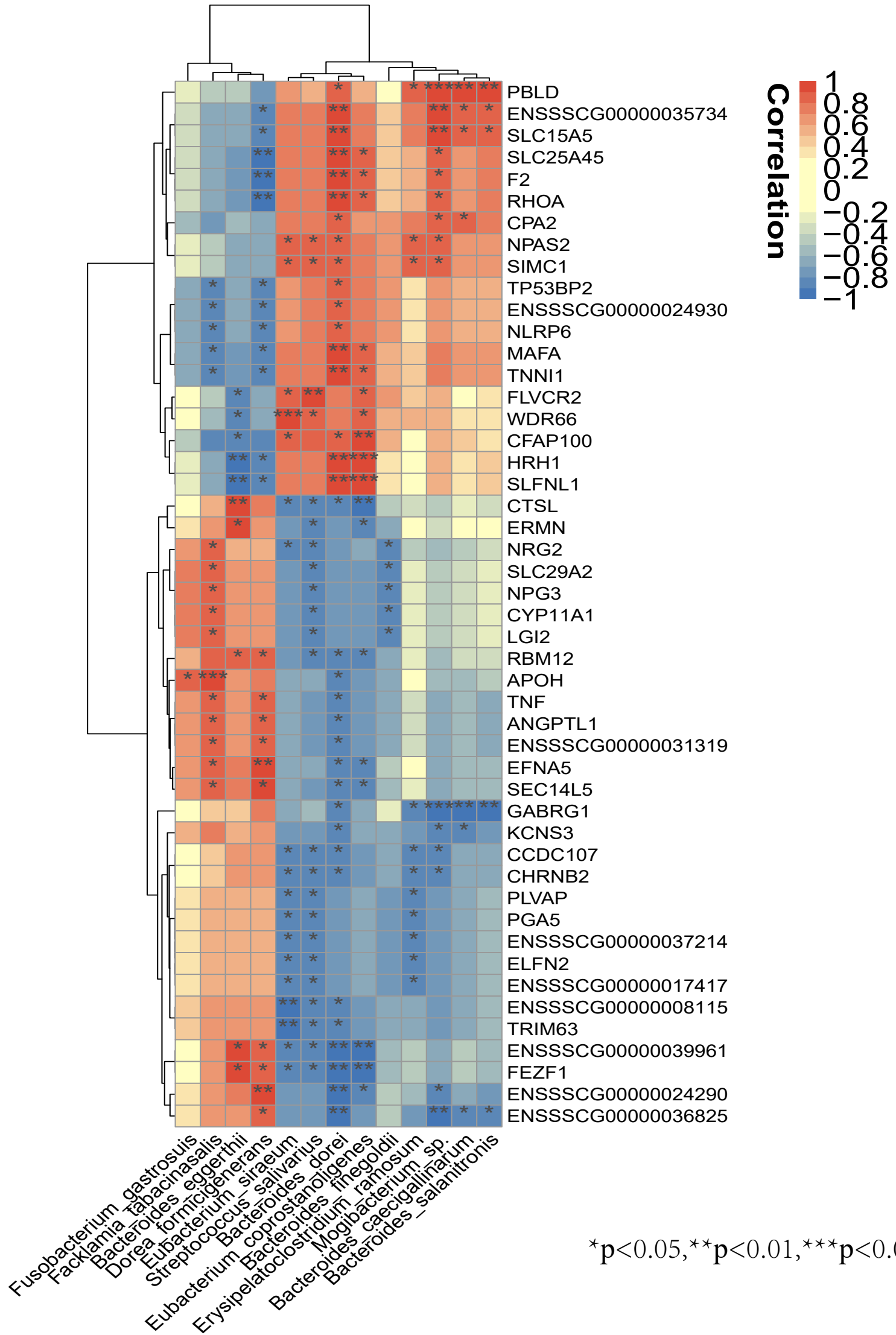

Supplement: Supplementary file 7 — Additional file 7. [file 12866_2023_3011_MOESM7_ESM.pdf]
