# Supplementary material for: Holo-omics analysis reveals the influence of gut microbiota on obesity indicators in Jinhua pigs
Source: BMC Microbiol. 2023 Nov 3;23:322. doi: 10.1186/s12866-023-03011-8 (PMC10623862; doi:10.1186/s12866-023-03011-8)

Profiles of differentially expressed genes and function in small and large intestinal segments

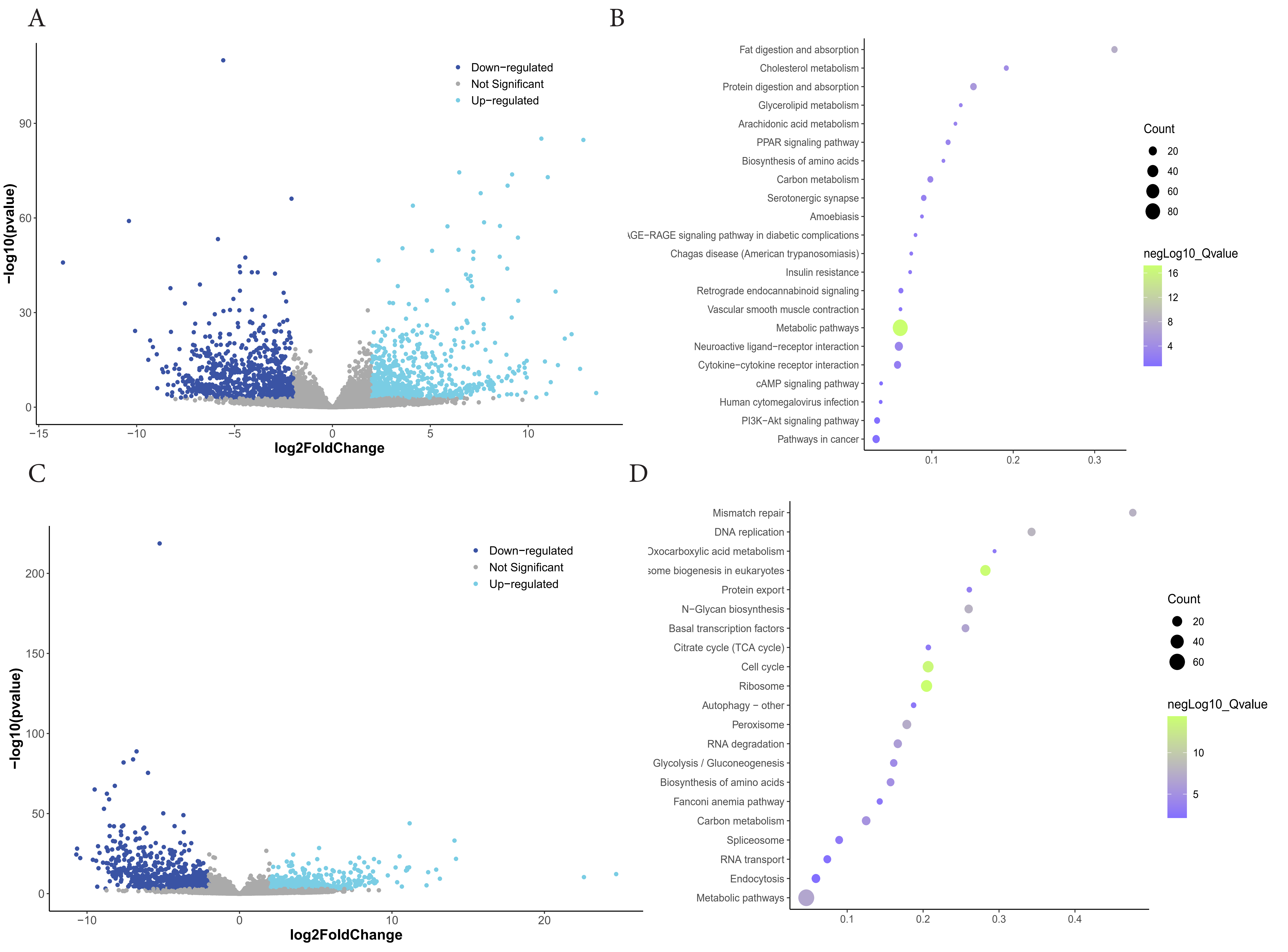

Supplement: Supplementary file 4 — Additional file 4. [file 12866_2023_3011_MOESM4_ESM.pdf]
